# Identification of (−)(E)-*N*-[2(*S*)-Hydroxy-2-(4-hydroxyphenyl) ethyl]ferulamide, a Natural Product Isolated from *Croton Pullei*: Theoretical and Experimental Analysis

**DOI:** 10.3390/ijms12129389

**Published:** 2011-12-15

**Authors:** Silvana de O. Silva, Rosana N.S. Peixoto, José Rogério A. Silva, Cláudio N. Alves, Giselle M.S.P. Guilhon, Lourivaldo S. Santos, Davi do S.B. Brasil

**Affiliations:** 1 Faculty of Chemistry, Institute of Exact and Natural Sciences, Federal University of Pará, CP 11101, Belém, PA 66075-110, Brazil; E-Mails: silvana.silva@icen.ufpa.br (S.O.S.); rspeixoto86@hotmail.com (R.N.S.P.); rogerio@ufpa.br (J.R.A.S.); giselle@ufpa.br (G.M.S.P.G.); lss@ufpa.br (L.S.S.); 2 Faculty of Chemistry Engineering, Institute of Technology, Federal University of Pará, Belém, PA 66075-110, Brazil

**Keywords:** DFT, B3LYP, B3PW91, NMR, IR, ferulic acid derivative

## Abstract

Ferulic acid (FA) and its derivatives (FADs) are known for a variety of biological activities, such as photo-protective agent, antioxidant, antiatherogenic and antiplasmodial activities. During structural definition of a FAD isolated from *Croton pullei*, the possibility of a heterologous series made this definition difficult. In this regard, computational simulations were performed using theoretical calculations at DFT level to predict Infrared (IR) and Nuclear Magnetic Resonance (NMR) data. The IR and NMR ^13^C and ^1^H data were compared with the theoretical calculations performed for three structural possibilities of a heterologous series. The theoretical results were compared with the experimental data through linear regression in order to define the most probable structure and showed satisfactory values.

## 1. Introduction

Ferulic acid (FA) and its derivatives (FADs) have been isolated from in a vast number of plant species. FA is a phenolic compound found in plant cell wall components [1], especially in wheat, corn, rice, tomatoes, spinach, cabbage and asparagus [2]. It has a variety of biological activities, such as photo-protective agent [3] and antioxidant [4]. Additionally, some ferulic ester dimers are potential antiatherogenic and antiplasmodial agents [5].

Analysis of pure substances using nuclear magnetic resonance (NMR) spectroscopy is one of the steps for obtaining more information about the chemical nature of known or unknown organic compounds [6]. Due to the versatility of NMR techniques, as well as the amount of information that can be extracted from analyzing NMR spectra, such information is extremely important in natural product chemistry [6], the area where this tool is most applicable.

**Figure 1:**
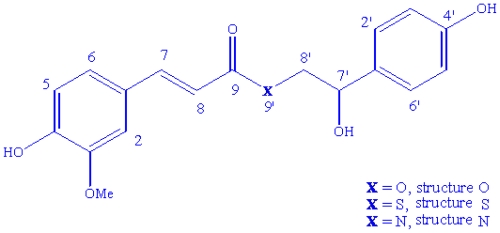
Planar visualization of **O**, **S** and **N** structure.

Complementary to NMR techniques, molecular modeling has appeared as an important set of computational tools for constructing, editing, visualizing and analyzing structural [7] of large [8,9] and small [10–13] molecular systems. Several recent papers have been published comparing data from experimental NMR with theoretical calculations performed using various computational models. Published work comparing theoretical and experimental data may be found for julocrotine [14], also isolated from *Croton pullei* (Euphorbiaceae) [15]. Besides julocrotine, the chemical investigation of *C. pullei* led to identification of the alkaloids crotonimides A and B, together with the terpenoids, lupeol, ribenone, sitosterol, kaurenoic acid, stigmasterol, among other compounds [15,16]. The chemical study of *C. pullei* was retaken and another substance was isolated and identified as a FAD but the heteroatom of the position 9′ was not identified by experimental NMR techniques. NMR data points to three structural possibilities with different heteroatom in position 9′: oxygen (an ester) [17], nitrogen (an amide) [18–20] or sulfur (a tioesther) (Figure 1). Thus, computational methods were used as an auxiliary tool for the elucidation of the structure of isolated natural products. All three structural possibilities were submitted to theoretical calculations using Density Functional Theory (DFT) [21]. Theoretical chemical shifts were compared with experimental data using linear regression, in order to define the heteroatom.

## 2. Results and Discussion

### 2.1. Conformational Analysis and Geometric Data

In Table S1 (see supplementary material) were presents the results to conformational analysis carried out to the **O**, **S** and **N** structures. The dihedral angle analyzed was C1′-C7′-C8′-X (X = O, S or N) for the three molecular structures. These results indicate that the conformers to each case have similar energies, thus we have selected two conformational structures to each compound (**O**, **N** and **S**) to start DFT calculations.

The data referring to the binding angles, binding distances and dihedral angles calculated using the B3LYP/6-31G(d,p) and B3LYP/6-31+G(d,p) methods (Table S2) after geometry optimization were analyzed to find the most probable heteroatom. As shown in Table S2, the structures calculated using B3LYP/6-31G(d,p) and B3LYP/6-31+G(d,p) showed very close bond lengths and bond angles for both conformers of structures **O**, **S** and **N**, however there are differences on the structural parameters between the three possibilities that are relevant especially near the **O**, **S** or **N** heteroatoms, as expected. The dihedral angle C1′-C7′-C8′-X obtained for the three structures possibilities (**O**, **S** and **N** structures) showed a difference of about 110° when calculated using B3LYP/6-31G(d,p) and B3LYP/6-31+G(d,p). While the calculated dihedral angle O7′-C7′-C8′-X varied significantly with the geometry change, the calculated dihedral angles C8′-X-C9-O9 e X-C9-C8-C7 were quite close. The dihedral angles C9-C8-C7-C1, C2-C3-O3-CMe and O4-C4-C3-O3 showed that the conformers are more sensitive to the geometry changes than to the applied DFT methods. So both methods can be used to describe the geometry of these molecules.

### 2.2. NMR Spectra and Statistical Analysis

The data for the FA, FADs and TMS (internal standard) (shielding constants of 32.1843 to ^1^H and 186.3296 to ^13^C) were calculated in gas phase at the B3PW91/DGDZVP and B3LYP/6-31+G(d,p) levels. The experimental and theoretical chemical shift for the ^13^C and ^1^H NMR data (chemical shifts) of the six structural possibilities of the FADs are showed in Tables 1 and 2 respectively, as well as the residue (RS) in ppm for each of the carbon and hydrogen atoms present in the structures. Tables 1 and 2 show the proximity existing between the values calculated by the DFT methodologies and those obtained experimentally for the **O**, **S** and **N** structures (Figures 1 and S1), which confirms the effectiveness of the computational model utilized for analyzing the possible structures. Consequently, the residual values in the structural region near the possible heteroatom provide an indication of which heteroatom may be the ligand of the studied FAD. This study showed that the proposed method can be used to identify unknown derivatives by the comparison between experimental and theoretical spectra. An example was carried out for the FA, confirming the effectiveness of computational methods (see Tables 3 and 4).

**Figure 2:**
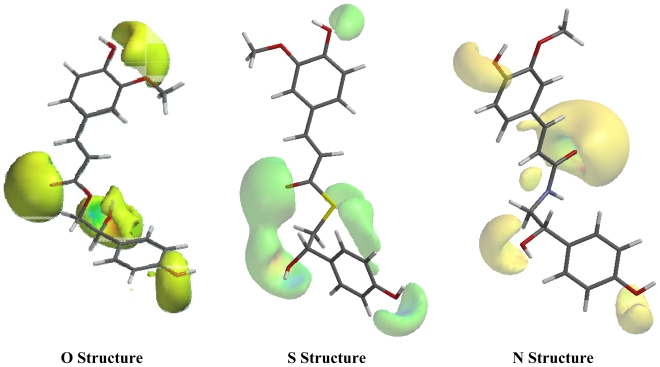
Electrostatic potential surfaces of the **O**, **S** and **N** structures optimized in B3LYP/6-31G(d,p).

The structures with the nitrogen atom shows lower residue values for δ(^13^C) than those of the **O** and **S** structures. Residue values (RS) of 7.2; 1.9 ppm (B3PW91/DGDZVP) and 5.2; 7.2 ppm [B3LYP/6-31+G(d,p)], respectively were obtained for positions 8′ and 9, while for the same positions, the **O** and **S** structures showed RS = 26.9; 0.3 ppm (B3PW91/DGDZVP) and RS = 26.5; 2.3 ppm [B3LYP/6-31+G(d,p)] for carbon 8′, respectively, and for position 9 values of 4.7; 30.6 ppm (B3PW91/DGDZVP) and 5.9; 23.0 [B3LYP/6-31+G(d,p)], were found respectively. To explain the high residual values at position 8′ of the **O** structure (26.9 and 26.5 ppm) and at position 9 for the **S** structure (30.6 and 23.0 ppm) two electronic effects can be used. The first is the electronegativity difference between the three suggested heteroatoms: the oxygen is the most electronegative atom and deshields the carbon atom at position 8′, consequently, it will display the largest δ. The second is the electronic effect of resonance between the pair of free electrons of the suggested heteroatom with the carbonyl group in position 9. In the case of **S** structure, the atom is too large and the orbital overlap necessary for the resonance effect to take place, it is more difficult, leading to an electron shielding effect on the carbon atom of position 9, reveled in the largest δ (Figure 2). As expected, hydrogens of position 8′ of O structure are more deshielded by the heteroatom than those of **N** and **S** structures.

The indication of the **N** structure has also been suggested by analysis of the linear fit parameters presented in Table 4. The correlation coefficients (*R*^2^) acquired for δ(^13^C) were 0.99 [B3PW91/DGDZVP and B3LYP/6-31+G(d,p)] and 0.94 (B3PW91/DGDZVP) for δ(^1^H) for the **N** structure and these values are higher than those found for the other structures. The MAE (mean absolute error) for the three structures was significantly corrected by the method employed. The general correlation of the data of Table 4 is satisfactory, given that all the signals coming from the different functional groups are divided into their own distinct regions. Thus, the general reliability of those calculations is confirmed

**Figure 3:**
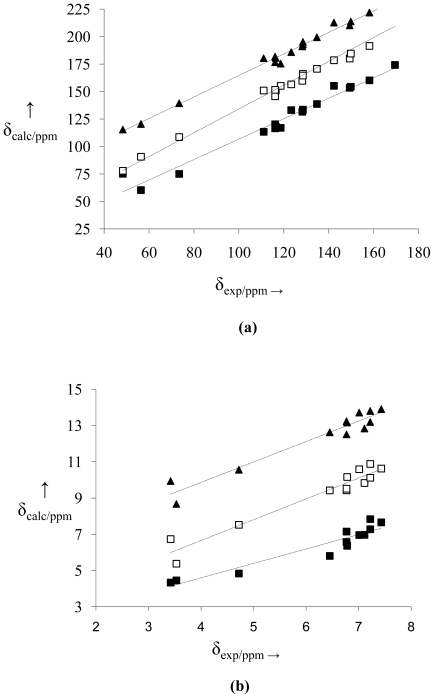
Correlation between experimental and calculated (B3PW91/DGDZVP) chemical shifts of ^13^C (**a**) and ^1^H (**b**) for structures **O** (■), **S** (□) and **N** (▴). NMR data of the structures **S** and **N** were displaced from 30 to 60 ppm (^13^C NMR) and from 3 to 6 ppm (^1^H NMR), respectively. For each set of data the linear fitting is also reported as a dashed line.

**Figure 4:**
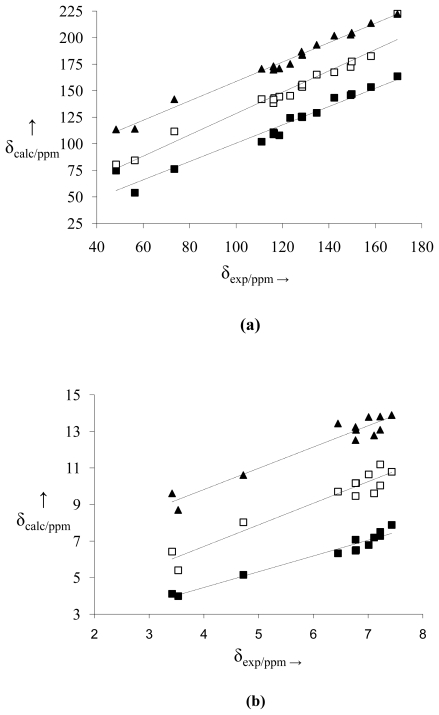
Correlation between experimental and calculated [B3LYP/6-31+G(d,p)] chemical shifts of ^13^C (**a**) and ^1^H (**b**) for structures **O** (■), **S** (□) and **N** (▴). NMR data of the structures **S** and **N** were displaced from 30 to 60 ppm (^13^C NMR) and from 3 to 6 ppm (^1^H NMR), respectively. For each set of data the linear fitting is also reported as a dashed line.

The linear setting performed with the experimental chemical shift data and calculated using the B3PW91/DGDZVP and B3LYP/6-31+G(d,p) methods for the three structural possibilities may be observed in Figures 3 and 4, respectively, where the linear correlation graphs for NMR data for ^13^C (a) and ^1^H (**b**) are presented. Among the NMR data for ^13^C and ^1^H of the three cases, the **N** structure is the best linearly adjusted one, including in the region where the electronic cloud is most dense. Statistical analysis shows that all models of ^1^H and ^13^C presented good linear regression (90% < *R*^2^ < 99%) (Table 4). However, the best models for the cross-evaluation procedure were in calculating the **N** structure for ^13^C NMR in B3LYP/6-31+G (d,p) (*F* = 1269.99; *s* = 3.4479; *s*_PRESS_ = 1.01 and *Q*^2^ = 98.3%), and ^13^C NMR in B3PW91/DGDZVP (*F* = 1675.82; *s* = 3.2676; *s*_PRESS_ = 0.89 and *Q*^2^ = 98.86%).

### 2.3. Infrared Spectrum

Frequency calculations of the normal vibration modes of the proposed structures were performed with the B3LYP/6-31G(d,p) and B3LYP/6-31+G(d,p) methods and the values obtained for the main absorption bands are showed in Table 5. A large absorption band was detected in 3370 cm^−1^ characterizing the presence of hydrogen bonds (OH and NH groups) in the experimental spectrum. The value obtained utilizing theoretical calculations for NH were 3612 cm^−1^ [B3LYP/6-31G(d,p)] and 3825 cm^−1^ [B3LYP/6-31+G(d,p)]; the vibration of OH groups for **O**, **S** and **N** structures were observed in 3782–3823 cm^−1^ [B3LYP/6-31G(d,p)] and 3828–3807 cm^−1^ [B3LYP/6-31+G(d,p)], 3760–3822 cm^−1^ [B3LYP/6-31G(d,p)] and 3828–3633 cm^−1^ [ B3LYP/6-31+G(d,p)] and 3763–3822 cm^−1^ [B3LYP/6-31G(d,p)] and 3824–3771 cm^−1^ [ B3LYP/6-31+G(d,p)], respectively. The carbonyl stretching band was 1680 cm^−1^ and the values obtained theoretically were, respectively, 1794 cm^−1^ [B3LYP/6-31G(d,p)] and 1768 cm^−1^ [B3LYP/6-31+G(d,p)] (**O** structure), 1754 cm^−1^ [B3LYP/6-31G(d,p)] and 1701 cm^−1^ [B3LYP/6-31+G(d,p)] (**S** structure) and 1758 cm^−1^ [B3LYP/6-31G(d,p)] and 1727 cm^−1^ [B3LYP/6-31+G(d,p)] (**N** structure). The asymmetric deformation of CH_3_ groups, was 1510 cm^−1^, and the values obtained by the computational method were 1504 cm^−1^ [B3LYP/6-31G(d,p)] and 1509 cm^−1^ [B3LYP/6-31+G(d,p)], 1502 cm^−1^ [B3LYP/6-31G(d,p)] and 1509 cm^−1^ [B3LYP/6-31+G(d,p)] and 1501 cm^−1^ [B3LYP/6-31G(d,p)] and 1510 cm^−1^ [B3LYP/6-31+G(d,p)] for the **O**, **S** and **N** structures, respectively. The data acquired with the two methodologies for the possible **O**, **S** and **N** structures, as expected, are close, because they are in the same range of absorption in the infrared experimental spectrum.

### 2.4. Polarimetry

The [α]_D_^25^ = −16° obtained experimentally indicate that the enantiomer isolated has absolute configuration S in the position 7′. Similar result was obtained by Dellagreca *et al.* [18].

## 3. Experimental Section

### 3.1. Collection and Extraction

Stems of *C. pullei* (1.00 kg) were collected in the municipality of Peixe-boi (PA, Brazil) and identified by Ricardo Secco, a botanist at the Museu Paraense Emílio Goeldi (Belém-PA, Brazil). The stems was air dried, ground and extracted by percolation with hexane (7 days) and methanol (14 days), with filtrations every 3 or 4 days. The solutions were concentrated under vacuum in a rotary evaporator, resulting in hexane extract (0.65 g) and methanolic extract (80.00 g). Part of the methanolic extract (40.00 g) was submitted to partition with dichloromethane, ethyl acetate and *n*-butanol. The resulting solutions were concentrated in a rotary evaporator. The dichloromethane phase (4.80 g) was fractionated by column chromatography (CC) in silica, using mixtures of hexane, ethyl acetate and methanol in gradients of increasing polarities as eluents. The column fraction eluted with the mixture of hexane-AcOEt 60% was submitted to column chromatography procedure on Sephadex LH-20 using methanol as eluent, leading to isolation of 35 mg of the FAD. [α]_D_^25^ = −16° (c 0.01, CH_3_OH), IR spectra were recorded in KBr in the spectrometer Nicolet IS10 FT-IR of Thermo Scientific) and ^1^H and ^13^C NMR data were obtained at 300 and 75.4 MHz, respectively, in CDCl_3_ using the solvent peak as the internal standard.

### 3.2. Computational Method

The **O**, **N** and **S** structures were drawn using HyperChem Release 7.5 software [23] and submitted to an initial optimization at PM3 [24]. In addition, the conformational analysis was carried out to confirm the minimum energy structure to the three possible structures (**O**, **N** and **S**), by carrying out a series of partial optimizations constraining the concerned dihedral angle step by step within the appropriate range, with a step size of 10°, these calculations were carried out using the HF/STO-3G basis set, the dihedral angle analyzed was C1′-C7′-C8′-X (X = O, S or N) for the three molecular structures. Previous studies about the importance of conformational analysis involving NMR calculations has been published [25,26] for a large number of natural products. The molecular structures were optimized with the Gaussian^®^ 03W [27] program, using the hybrid functional B3LYP together with the 6-31G(d,p) and 6-31G+(d,p) basis set. Vibrational analysis was performed using the procedure contained in the Gaussian^®^ 03W package [27] with the DFT method using the B3LYP/6-31G(d,p) and B3LYP/6-31+G(d,p) levels, in the gas phase. This ensured that each gradient optimization located indeed a true minimum energy structure (no imaginary frequencies). The normal vibration modes were visualized using the Hyperchem 7.5 program [23]. Data for NMR (^13^C and ^1^H chemical shifts) were calculated using the DFT/B3PW91/DGDZVP and B3LYP/6-31+G(d,p) methods in vacuum. Recently, we have successfully used DFT/B3PW91/DGDZVP methodology to study ^1^H and ^13^C NMR spectra of cordatin [28] and 8-epicordatin [29]. Two conformers each structure (**O**, **N** and **S**) were submitted to DFT calculations by different methodologies (see Table S3); we chose this methods because the energies of the conformers are similar. The Spartan ′08 program [30] was utilized to calculate electrostatic potential surfaces of the **O**, **S** and **N** structures utilizing the DFT/B3LYP/6-31G(d,p) method.

### 3.3. Statistical Analysis

MINITAB14 [31] software was employed for statistical analysis of NMR linear regression data. The correlation coefficients (*R*^2^), the Fischer values (*F*) and the standard deviation (*s*) were the statistical parameters chosen for this analysis. For each one of the conformer of the molecules studied (**O**, **N** and **S** structures) parameters are presented for linear adjustment *a* and *b*: δ_calc_ = *a + b*δ_exp_, mean absolute error: MAE = ∑|δ_calc_ – δ_exp_|/*n* and corrected mean absolute error: CMAE = ∑| δ_corr_ – δ_exp_|/*n* [32]. These parameters, calculated for experimental and theoretical data in these structures, allow the study of chemical displacements (ppm), as well as of residues: RS = |δ_exp_ – δ_calc_|of the hydrogen and carbon atoms and the influence of the heteroligands involved in the region next to positions 8 and 9′.

The equations obtained were tested for their predictive power using a cross-validation procedure, which is a practical and reliable method for testing significance. This approach, known as “leave on out”, consists in developing a number of models with one sample omitted at the time. After these models are obtained, the omitted data are predicted and the differences between the real and predictor values are calculated. The sum of the squares of this difference is computed, and finally, the performance of the model (its predictive capacity) can be given by PRESS (predicted sum of squares) and by *s*_PRESS_ (standard deviation of the cross-validation) [33]:

(1) $$\begin{array}{c} \text{PRESS}=\sum_{i=1}^{n}{\left(y_{i}-{\hat{y}}_{i}\right)}^{2}\\ s_{\text{PRESS}}=\frac{\sqrt{\text{PRESS}}}{n-k-1} \end{array}$$

where y is the experimental value, *ŷ* is the predictor value, *n* is the number of samples used for the construction model and *k* is the number of NMR parameters.

The predictive capacity [33] for the model was also quantified in terms of *Q*^2^, which is defined as:

(2) $$Q^{2}=1.0-\frac{\sum_{i=1}^{n}{\left(y_{i}-{\hat{y}}_{i}\right)}^{2}}{\sum_{i=1}^{n}{\left(y_{i}-{\bar{y}}_{i}\right)}^{2}}\;\text{where},\bar{y}=y_{mean}$$

## 4. Conclusions

Computational calculations performed at the DFT level for a heterologous series showed excellent results. The computational method together with the ^13^C and ^1^H NMR and polarimetric analysis confirmed that the ferulic acid derivative present in a the stems of *C. pullei* is (−)(E)-*N*-[2(*S*)-Hydroxy-2-(4-hydroxyphenyl)ethyl]ferulamide. Thus the heteroatom of position 9′ is a nitrogen atom (**N)**. The statistical parameters revealed that the B3PWP1/DGDZVP and B3LYP/6-31+G(d,p) methodology tested for FADs and FA offer good predictive capacity and good significance. The **N** structure present the vest values for *R*^2^, *F*, *s*_PRESS_ and *Q*^2^ after cross-evaluation to ^13^C NMR data.

## Figures and Tables

**Table 1:** Experimental ^13^C nuclear magnetic resonance (NMR) data (Exp.), calculated ^13^C NMR data (Calc.) with B3PW91/DGDZVP and residue (RS) in ppm for the **O**, **S** and **N** structures.

		B3PW91/DGDZVP						B3LYP/6-31+G(d,p)					
		
Position	Exp.	Structure O		Structure S		Structure N		Structure O		Structure S		Structure N	
		
		Calc.	RS	Calc.	RS	Calc.	RS	Calc.	RS	Calc.	RS	Calc.	RS
1	128.2	131.6	3.4	129.8	1.6	131.0	2.8	125.7	2.5	124.2	4.0	126.8	1.4
2	111.0	113.4	2.3	121.0	10.0	120.3	9.3	101.9	9.0	112.1	1.1	110.7	0.3
3	149.3	153.5	4.2	150.2	0.8	150.2	0.9	145.9	3.4	142.5	6.9	142.8	6.4
4	149.8	154.4	4.6	154.7	4.9	153.7	3.9	146.9	2.9	147.5	2.3	144.6	5.2
5	116.4	118.3	1.9	120.9	4.5	120.6	4.2	110.2	6.2	113.2	3.2	111.9	4.5
6	123.3	133.2	9.9	126.6	3.3	125.9	2.6	124.4	1.1	115.3	8.0	115.2	8.1
7	142.3	155.2	12.9	148.5	6.2	152.7	10.4	143.5	1.2	137.5	4.8	141.8	0.5
8	118.6	117.0	1.6	125.3	6.7	115.4	3.2	108.0	10.6	114.4	4.2	110.9	7.6
9	169.5	174.2	4.7	200.1	30.6	171.4	1.9	163.6	5.9	192.5	23.0	162.3	7.2
1′	134.7	138.7	4.0	140.7	6.0	139.4	4.7	129.2	5.5	135.2	0.5	133.3	1.4
2′	128.5	133.6	5.1	136.0	7.5	132.9	4.4	125.8	2.7	123.6	4.9	124.1	4.4
3′	116.1	120.3	4.2	115.7	0.4	121.8	5.7	110.9	5.1	108.4	7.7	113.0	3.1
4′	158.1	160.3	2.3	161.6	3.4	161.9	3.8	153.4	4.7	152.6	5.5	153.7	4.4
5′	116.1	116.6	0.5	121.6	5.5	116.7	0.6	108.9	7.2	111.8	4.3	109.7	6.4
6′	128.5	133.4	4.9	134.6	6.1	135.2	6.7	125.0	3.5	125.8	2.8	123.6	4.9
7′	73.4	75.1	1.7	78.6	5.2	79.4	5.9	76.2	2.8	81.7	8.3	81.9	8.6
8′	48.3	75.3	26.9	47.9	0.3	55.5	7.2	74.8	26.5	50.6	2.3	53.5	5.2
OMe	56.4	60.7	3.9	60.7	4.3	60.5	4.1	53.9	2.51	54.4	2.0	54.0	2.4

RS = |δ_exp_ – δ_calc_|

**Table 2:** Experimental ^1^H NMR data (Exp.), calculated ^1^H NMR data (Calc.) with B3PW91/DGDZVP and residue (RS) in ppm for the **O**, **S** and **N** structures.

		B3PW91/DGDZVP						B3LYP/6-31+G(d,p)					
		
Position	Exp.	Structure O		Structure S		Structure N		Structure O		Structure S		Structure N	
		
		Calc.	RS	Calc.	RS	Calc.	RS	Calc.	RS	Calc.	RS	Calc.	RS
2	7.11	6.97	0.14	6.83	0.28	6.83	0.28	7.19	0.08	6.61	0.50	6.78	0.33
5	6.78	6.37	0.41	7.16	0.38	7.17	0.39	6.51	0.27	7.17	0.39	7.08	0.30
6	7.01	6.96	0.05	7.59	0.58	7.71	0.70	6.78	0.23	7.65	0.64	7.79	0.78
7	7.43	7.66	0.23	7.63	0.20	7.90	0.47	7.88	0.45	7.79	0.36	7.89	0.46
8	6.45	5.81	0.64	6.42	0.03	6.62	0.17	6.33	0.12	6.71	0.26	7.43	0.98
2′	7.22	7.28	0.06	7.12	0.10	7.80	0.58	7.28	0.06	7.04	0.18	7.81	0.59
3′	6.77	7.15	0.38	6.43	0.34	7.23	0.46	7.08	0.31	6.47	0.30	7.24	0.47
5′	6.77	6.57	0.20	7.21	0.44	6.51	0.26	6.47	0.30	7.17	0.40	6.52	0.25
6′	7.22	7.84	0.62	7.88	0.66	7.19	0.03	7.51	0.29	8.20	0.98	7.09	0.13
7′	4.72	4.84	0.12	4.52	0.20	4.56	0.16	5.15	0.43	5.03	0.31	4.61	0.11
8′ a	3.53	4.46	0.93	2.38	1.15	2.67	0.86	3.99	0.46	2.40	1.13	2.70	0.83
8′ b	3.42	4.33	0.91	3.73	0.31	3.94	0.52	4.11	0.69	3.43	0.01	3.61	0.19

RS = |δ_exp_ – δ_calc_|

**Table 3:** Experimental [22] ^1^H and ^13^C NMR data, calculated ^1^H NMR data (Calc.) with B3LYP/6-31+G(d,p) and residues (RS) in ppm for the FA.

Position	^13^C			^1^H		

	Exp. [22]	Calc.	RS	Exp. [22]	Calc.	RS
1	127.8	126.1	1.6	-	-	
2	111.6	113.5	1.8	7.2	6.7	0.5
3	151.5	145.1	6.4	-	-	
4	149.9	145.7	4.2	-	-	
5	116.5	111.4	5.1	6.8	6.6	0.2
6	123.9	113.6	10.4	7.5	7.5	0.4
C-α	115.9	107.7	8.2	6.3	6.5	0.2
C-β	146.9	147.2	0.3	7.6	7.8	0.2
C=O	171.2	162.7	8.5	-	-	
OMe-3	56.5	53.9	2.5	3.9	3.8	0.1

RS = |δ_exp_ – δ_calc_|

**Table 4:** Correlation and linear adjustment parameters for the NMR properties of the **O**, **S** and **N** structures and ferulic acid (FA).

	δ	*a*	*b*	*R*^2^	MAE	CMAE	*s*	PRESS	*s*_PRESS_	*F*	*Q*^2^
B3PW91/DGDZVP	δ(^1^H) O	0.80	1.41	0.90	0.39	0.40	0.4910	3.6794	0.19	86.11	84.12%
	δ(^1^H) S	1.16	−0.98	0.94	0.39	0.31	0.4182	3.0896	0.18	122.49	86.67%
	δ(^1^H) N	1.13	−0.64	0.94	0.41	0.31	0.3856	2.6661	0.16	145.83	88.50%
	δ(^13^C) O	0.93	13,87	0.96	5.49	4.32	6.3579	959.879	1.94	430.88	94.69%
	δ(^13^C) S	1.08	−4.11	0.97	5.97	3.53	5.7831	913.886	1.89	524.12	94.94%
	δ(^13^C) N	0.98	6.86	0.99	4.57	2.21	3.2676	205.189	0.89	1675.82	98.86%

B3LYP/6- 31+G(d,p)	δ(^1^H) O	0.86	1.00	0.96	0.31	0.25	0.2786	1.0415	0.10	222.41	94.23%
	δ(^1^H) S	1.18	−1.02	0.92	0.46	0.35	0.5231	4.6355	0.22	118.15	86.78%
	δ(^1^H) N	1.16	−0.83	0.93	0.45	0.32	0.4879	3.8216	0.19	131.42	88.65%
	δ(^13^C) O	0.86	14.22	0.95	5.74	5.42	6.7773	1331.07	2.28	293.17	90.63%
	δ(^13^C) S	1.00	−1.92	0.95	5.32	4.70	7.4911	1304.14	2.26	324.63	93.18%
	δ(^13^C) N	0.91	7.30	0.99	4.56	2.82	3.4479	260.415	1.01	1269.99	98.30%
	δ(^1^H) FA	1.02	−0.12	0.95	0.26	0.24	0.3601	0.8689	0.23	71.56	91.13%
	δ(^13^C) FA	0.97	−0.28	0.98	4.90	3.12	4.0927	179.844	1.68	498.65	97.88%

**Table 5:** Experimental and theoretical attribution of absorption bands in the IR (in cm^−1^) calculated at the B3LYP/6-31G(d,p) level for the heterologous series.

Vibrational Mode (cm^−1^)	Experimental	Theoretical—B3LYP/6-31G(d,p)			Theoretical—B3LYP/6-31+G(d,p)		

		O structure	S structure	N structure	O structure	S structure	N structure
*υ* (O-H aliphatic)	3370	3782	3808	3810	3806	3633	3825
*υ* (O-H aromatic)	3370	3818	3760	3763	3822	3765	3612
*υ* (N-H)	3370	-	-	3612	-	-	3612
*υ* (O-H aromatic)	3370	3822	3822	3822	3828	3828	3770
*υ*_a_ (CH_2_) + *υ*_s_ (C-H sp^2^)	-	3128	3156	3127	3130	3137	3114
*υ_s_* (CH_2_) + *υ*_s_ (C-H sp^3^) + *υ*_s_ (C-H sp^2^)	-	3077	3089	3076	3081	3098	3094
*υ*_a_ (CH_3_) + *υ* (C-H sp^2^)	-	3154	3157	3155	3157	3159	3157
*υ* (C-H sp^2^) + *υ*_a_ (C-H sp^2^ aromatic)	-	3207	3204	3206	3207	3206	3201
*υ*_s_ (CH_3_)	-	3016	3026	3025	3017	3029	3027
*υ* (C-H sp^3^) + *υ*_s_ (CH_2_)	2930	3038	3018	2985	3073	3075	3064
δ_a_ (C-H sp^2^ aromatic) + *υ_s_* (C-O)	-	1675	1673	1673	1664	1662	1662
*υ* (C=O) + *υ* (C=C trans) + δ(C-H) [+δ(N-H)] a	1680	1794	1755	1758	1768	1701	1727
*υ*_s_ (C=C trans) + *υ* (C=O) + δ (C-H sp^2^) or [+ δ(N-H)] a	1600	1691	1755	1685	1679	1754	1674
δ_a_ (CH_3_)	1510	1505	1502	1502	1509	1509	1510
δ_s_ (C-H sp^2^ aromatic) + *υ* (C-O secondary alcohol)	1480	1307	1307	1309	1311	1309	1308
δ_s_ (C-H sp^2^) + *υ* (C-O secondary alcohol)	1390	1319	1316	1319	1319	1327	1318
δ_s_ (C-H sp^2^) + δ_a_ (CH_3_)	1110	1225	1220	1179	1220	1228	1227

*υ*: stretching; *υ*_a_: asymmetrical stretching; *υ_s_*: symmetrical stretching; δ: deformation; δ_a_: asymmetrical deformation; δ_s_: symmetrical deformation;

a Only for **N** structure.
